# Intelligence

**Published:** 1807-11

**Authors:** 


### INTELLIGENCE.

M. Jouville, the only mineralogist in the island of Ceylon, has transmitted to Dr. De Carro, of Vienna, some interesting observations on its mineralogy. From these it appears, that no gems have been discovered in their mattrices; all that he ever saw having been found in currents, and no others are in that market. It appears that the king of Candy is averse to permitting Europeans to explore his mountains; and, on the other hand, they are so thickly covered with the vegetation of ages, that no fissures are to be seen, by which the mineralogist can be directed. The Candians take no further trouble to search for stones or minerals, than raking for them in the beds of currents, after the rainy season.

Besides the ruins in the Illinois and Wabash countries, of which accounts have frequently been given, there are others not less remarkable, several hundred miles farther west; particularly in the country about the great falls of the Mississippi. On approaching these falls, called St. Anthony's, pyramids of earth are frequently met with, from thirty to seventy, and even eighty feet, in height. These are, most probably, the tombs of the ancient kings and chieftains of this part of America; though there are others, which are thought to have been erected in consequence of some signal victory, and possibly to cover the bodies of those who were slain. In digging horizontally into several of these pyramids, a little above the base, there is generally found a stratum of a white substance, somewhat like moist lime, and rather glutinous, extending, in all probability, several yards within, or, perhaps, nearly the whole length of the diametrical line. There is every reason to believe this consolidated substance to be the remains of skeletons buried twenty centuries ago, and converted, by time and the operations rations of natural causes, into their present state. Many tokens remain, on both sides of the Mississippi, of their being, in ancient ages, as well cultivated and as thickly inhabited, as the country on the Danube or the Rhine; and which sufficiently prove that the denominating of America a new world, has been an opinion too hastily taken up. A copper-mine was opened, some years since, further down the Mississippi; and, to the great surprise of the labourers, a large collection of the mining tools were found, several fathoms below the superfices of the earth. Another person, in digging for a well, discovered a furnace of brick-work, five fathoms below the present surface; and in this furnace were found a quantity of coals and burnt wood. Not long since, at a spot on the Ohio, where the banks had been watered by the undermining of the water, a stone dropped out, of the harder kind of black marble, about seven pounds in weight, having twelve equal surfaces, each surface being mathematically equilateral and equiangular five-sided figures. Near the falls of the Mississippi there is a salt spring, in the bed of the river, which has been inclosed with stone-work of unknown antiquity, to keep out the fresh water. In times of freshes, however, the river overflows the stone-work, and mixes with the brine, so that it does not afford salt to the savages hereabout, until the river is considerably fallen. In several places, circular fortifications have been discovered in the same country; these are constantly enclosed with deep ditches, and fenced with a breast-work. Some other facts will appear soon on this subject, in the travels of Mr. Aske into those countries.

Mr. Samuel Young, of the London College of Surgeons, &c. has a work in the press, entitled, A Course of Lectures, addressed to the Students in Surgery; comprising a Systematic Reform of the Modern Practice of Adhesion, particularly in Relation to the Abuses of the Thread Suture, in the Surgery of Wounds.

